# Supplementary material for: Social support and health related quality of life among older people in covid-19 pandemic: The mediating role of resilience
Source: PLoS One. 2026 Jun 5;21(6):e0342982. doi: 10.1371/journal.pone.0342982 (PMC13240856; doi:10.1371/journal.pone.0342982)
Supplement: S1 File — (PDF) [file pone.0342982.s001.pdf]

Tabulation of sex

	Freq.	Percent	Cum.
Male	247	72.22	72.22
Female	95	27.78	100.00
Total	342	100.00	

Tabulation of agegr

	Freq.	Percent	Cum.
60-65	73	21.35	21.35
65-70	76	22.22	43.57
70-75	82	23.98	67.54
75-80	63	18.42	85.96
80-85	38	11.11	97.08
>85	10	2.92	100.00
Total	342	100.00	

Tabulation of job

	Freq.	Percent	Cum.
آزاد	60	17.54	17.54
بازنشسته	169	49.42	66.96
بیکار	73	21.35	88.30
نظامی	6	1.75	90.06
کشاورز	34	9.94	100.00
Total	342	100.00	

Descriptive Statistics

Variable	Obs	Mean	Std. Dev.	Min	Max
Resilience	342	58.237	11.311	16	94
SocialS	342	78.333	12.218	26	106
QoL	342	93.14	9.502	65	121

-> sex = Male

Variable	Obs	Mean	Std. Dev.	Min	Max
Resilience	**247**	**59.5668**	**10.84867**	**16**	**86**
SocialS	**247**	**80.11741**	**12.42598**	**26**	**106**
QoL	**247**	**92.72065**	**10.16405**	**66**	**121**

-> sex = Female

Variable	Obs	Mean	Std. Dev.	Min	Max
Resilience	**95**	**54.77895**	**11.80442**	**24**	**94**
SocialS	**95**	**73.69474**	**10.35957**	**52**	**98**
QoL	**95**	**94.23158**	**7.449765**	**65**	**117**

-> job2 = 1

Variable	Obs	Mean	Std.Dev.	Min	Max
Resilience	60	55.267	8.731	36	74
SocialS	60	74.567	11.775	44	99
QoL	60	94.050	7.621	74	113

-> job2 = 2

Variable	Obs	Mean	Std.Dev.	Min	Max
Resilience	169	59.621	11.180	24	86
SocialS	169	81.059	11.545	46	106
QoL	169	93.379	9.922	67	119

-> job2 = 3

Variable	Obs	Mean	Std.Dev.	Min	Max
Resilience	73	57.260	12.838	16	94
SocialS	73	76.192	13.300	26	98
QoL	73	90.479	10.086	65	121

-> job2 = 4

Variable	Obs	Mean	Std.	Dev.	Min	Max
Resilience	6	53.167	3.656	49	58	
SocialS	6	66.167	11.771	44	77	
QoL	6	94.500	6.253	87	104	

-> job2 = 5

Variable	Obs	Mean	Std.Dev.	Min	Max
Resilience	34	59.588	12.376	31	86
SocialS	34	78.176	10.400	41	95
QoL	34	95.824	8.653	72	109

-> agegr = 60-65

Variable	Obs	Mean	Std.Dev.	Min	Max
Resilience	73	61.918	11.150	24	94
SocialS	73	81.671	13.096	41	100
QoL	73	93.370	10.469	69	112

-> agegr = 65-70

Variable	Obs	Mean	Std.Dev.	Min	Max
Resilience	76	60.500	10.944	28	86
SocialS	76	79.618	11.644	49	106
QoL	76	93.474	10.104	65	121

-> agegr = 70-75

Variable	Obs	Mean	Std.Dev.	Min	Max
Resilience	82	57.793	9.264	36	72
SocialS	82	77.512	11.043	49	97
QoL	82	93.683	9.462	66	117

-> agegr = 75-80

Variable	Obs	Mean	Std.	Dev.	Min	Max

Resilience	63	55.651	11.374	29	80
SocialS	63	76.746	10.546	46	99
QoL	63	93.222	7.544	76	113

-> agegr = 80-85

Variable	Obs	Mean	Std.Dev.	Min	Max
Resilience	38	52.500	10.150	25	77
SocialS	38	74.105	12.907	47	94
QoL	38	91.737	8.601	73	103

-> agegr = >85

Variable	Obs	Mean	Std.	Dev.	Min	Max
Resilience	10	55.900	20.507	16	74	
SocialS	10	77	19.994	26	92	
QoL	10	89.300	12.746	67	111	

Linear regression

resis_total	Coef.	St.Err.	t-value	p-value	[95% Conf	Interval]	Sig
SocialS	.445	.044	10.19	0	.359	.53	***
sex	-2.039	1.176	-1.73	.084	-4.352	.274	*
agegr	-1.386	.371	-3.73	0	-2.117	-.656	***
job2	.704	.464	1.51	.131	-.21	1.617	
Constant	28.293	4.453	6.35	0	19.534	37.052	***

Mean dependent var	58.237	SD dependent var	11.311
R-squared	0.316	Number of obs	342
F-test	38.928	Prob > F	0.000
Akaike crit. (AIC)	2508.852	Bayesian crit. (BIC)	2528.026

*** *p<.01*, ** *p<.05*, * *p<.1*

Linear regression

quality3	Coef.	St.Err.	t-value	p-value	[95% Conf	Interval]	Sig
SocialS	.246	.042	5.85	0	.163	.329	***
sex	3.087	1.133	2.72	.007	.859	5.315	***
agegr	-.046	.358	-0.13	.899	-.75	.658	
job2	.025	.447	0.05	.956	-.856	.905	

Constant	70	4.29	16.32	0	61.562	78.439	***

Mean dependent var	93.140	SD dependent var	9.502
R-squared	0.100	Number of obs	342
F-test	9.398	Prob > F	0.000
Akaike crit. (AIC)	2483.403	Bayesian crit. (BIC)	2502.577

*** *p<.01*, ** *p<.05*, * *p<.1*

Linear regression

quality3	Coef.	St.Err.	t-value	p-value	[95% Conf	Interval]	Sig
resis_total	.341	.044	7.66	0	.253	.428	***
sex	3.179	1.088	2.92	.004	1.039	5.318	***
agegr	.281	.353	0.80	.427	-.414	.975	
job2	-.202	.435	-0.47	.642	-1.057	.653	
Constant	68.926	3.599	19.15	0	61.847	76.005	***

Mean dependent var	93.140	SD dependent var	9.502
R-squared	0.156	Number of obs	342
F-test	15.575	Prob > F	0.000
Akaike crit. (AIC)	2461.555	Bayesian crit. (BIC)	2480.729

*** *p<.01*, ** *p<.05*, * *p<.1*

.

quality3	Coef.	Std. Err.	t	P>|t|	[95% Conf.	Interval]
resis_total	.2750752	.0503743	5.46	0.000	.1759865	.3741639
SocialS	.1236688	.0461531	2.68	0.008	.0328833	.2144543
sex	3.647657	1.092111	3.34	0.001	1.499421	5.795893
agegr	.3358505	.3505397	0.96	0.339	-.3536784	1.025379
job2	-.1689597	.4309364	-0.39	0.695	-1.016633	.6787135
_cons	62.21771	4.357389	14.28	0.000	53.64651	70.78891

.

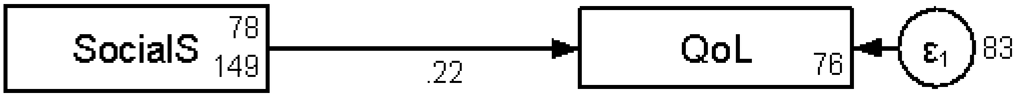
SocialS
78
149
.22
QoL
76
ε1
83

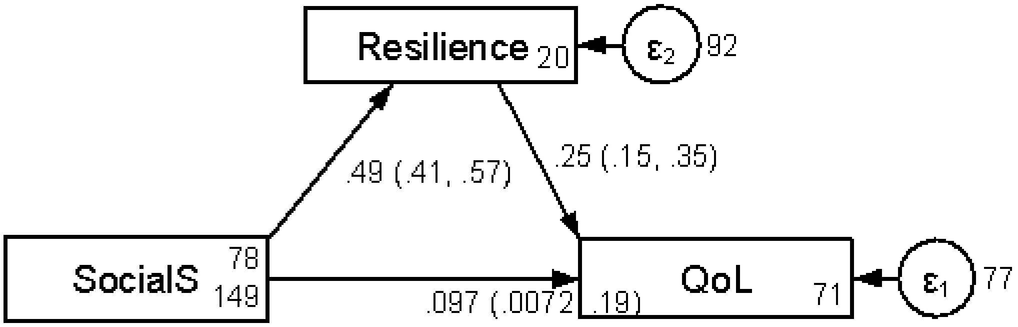
Resilience
20
ε2
92
.49 (.41, .57)
.25 (.15, .35)
SocialS
78
149
.097 (.0072, .19)
QoL
71
ε1
77
