# Supplementary material for: Social support and health related quality of life among older people in covid-19 pandemic: The mediating role of resilience
Source: PLoS One. 2026 Jun 5;21(6):e0342982. doi: 10.1371/journal.pone.0342982 (PMC13240856; doi:10.1371/journal.pone.0342982)
Supplement: Attachment — Submitted filename: PONE-D-24-51857.docx [file pone.0342982.s002.docx]

**Dear Editor**

**Thank you for the opportunity to review. The review of manuscript PONE-D-24-51857, entitled “Social Support and Health Related Quality of Life among Older People in Covid-19 Pandemic: The Mediating Role of Resilience” done and sent for you.**

**Comments to authors**

**Abstract**

- Several grammatical errors are present, such as line 42 "341 older people over 60 years were participated," which should be corrected to "341 older adults over 60 years participated".

- In line 39, Phrasing such as "it will be possible to improve…" is vague and speculative. A more assertive tone is recommended.

- In line 48, there are misplaced commas (e.g., β = 0.44,).

- In lines 48 and 49, the statistical findings are not contextualized—what does a β = 0.097 mean in practical terms for HRQoL?

- I recommended briefly mention prior studies and highlight the novelty of this research.

**Introduction**

- I would suggest to summarize the introduction section.

- Definitions and relationships between HRQoL and social support are repeated unnecessarily.

- The concept of resilience is introduced multiple times with overlapping definitions and citations, which disrupts the flow and dilutes impact.

- The paragraph transitions are abrupt, and the logical progression of ideas is weak. For example, demographic data about Iran’s aging population is inserted without a clear link to the preceding or following content.

- The final paragraph claims novelty in examining the three variables concurrently but does not adequately support this claim with references or a synthesis of existing gaps in the literature.

In line 100, the writing is overly formal and verbose in places, which may hinder readability. Phrases such as "engendered by the emergence of this recent epidemic" could be simplified.

**Materials and Methods**

- Please explain the demographic factors.

- In line 137, Please replace the word "Instrument" in this sentence with "The questionnaires".

- Authors should be summarized the parts mentioned in lines 138-169.

- The study uses cluster random sampling across 16 neighborhoods, but the rationale for selecting two neighborhoods per district is not fully explained.

- It is recommended to clarify how the sample size was determined and whether power analysis was conducted to justify the inclusion of 341 participants.

- The method of replacing non-respondents with others from the same age group and neighborhood may introduce selection bias so, it is recommended to discuss the potential impact of this substitution on sample representativeness and data validity.

- While reliability coefficients are reported for all instruments, the validity of the resilience questionnaire in the elderly population is not discussed. It is recommended to provide more detail on the cultural and age-specific validation of the Conner-Davidson resilience scale in Iranian elderly populations.

- The authors mention controlling for gender, age, and employment status, but do not specify how these were included in the regression or SEM models.

- Include a statement confirming ethical clearance from an institutional review board and the process for obtaining participant consent.

- Clarify whether missing data were present and how they were handled.

**Results**

- In lines 185 and 188, tables and 2 change to Tables 1 and 2.

- In line 206, the relationship between resilience and HRQoL is reported with two different β values (0.34 and 0.24), which creates confusion.

- Differences in mean scores between demographic groups (e.g., gender, employment status, age) are described, but no statistical tests (e.g., t-tests, ANOVA) are reported to determine significance.

- The scales used for resilience (out of 125), social support (out of 115), and HRQoL (out of 100) are mentioned, but the instruments are not identified.

- The phrase "considering demographic variables" is used repeatedly, but it is unclear which variables were controlled for and how they were incorporated into the models.

- Some reported β values (e.g., 0.097 for direct effect) are statistically significant but small. The practical relevance of these findings is not discussed.

- The finding that unemployed individuals had higher social support than employed ones contradicts common assumptions and prior literature.

**Discussion**

**-** The reported direct effect of social support on HRQoL (β = 0.04) is statistically significant but extremely small. The discussion does not address whether this effect is practically meaningful.

- While the authors cite numerous studies, the discussion lacks critical analysis of conflicting findings, methodological differences, or contextual limitations.

- The mediating role of resilience is reiterated multiple times without adding new insights or elaborating on mechanisms. It is recommended to consolidate repetitive statements and expand on how resilience functions as a mediator in practical terms.

- The manuscript suggests that findings can inform policy but does not specify how interventions might be designed or implemented. Offer concrete examples of resilience-building or social support strategies that could be applied in community or clinical settings.

**References**

- Please correct the errors in throughout the References. However, Authors must use the abbreviation for journal name. Articles rewrite according to "PLOS ONE" format.

- In line 355, "WH" change "WHO".

- Please delete "_⸋_" in line 377.

- In lines 406 and 409, articles with more than 6 authors must also rewrite according to "PLOS ONE" format.

- In line 423, please delete ".", ":" and ";".

- In line 424, Karimi Y KM HY should be corrected.

- 2006;5(1):0-. in line 431, rewrite.

- Please delete "," in line 434.

- In line 444, authors should be corrected.

- In line 450, please delete space and ",".

- In lines 466 and 472, authors should be corrected.

**Tables**

- Authors should explain "*****" in Table 5.

**With regards,**

**Afsaneh Amouei**
